# Mapping the Ligand-Binding Region of *Borrelia hermsii* Fibronectin-Binding Protein

**DOI:** 10.1371/journal.pone.0063437

**Published:** 2013-05-02

**Authors:** Christiane Brenner, Katharina Bomans, Jüri Habicht, Markus M. Simon, Reinhard Wallich

**Affiliations:** Infectious Immunology Group, Institute for Immunology, University of Heidelberg, Heidelberg, Germany; University of Kentucky College of Medicine, United States of America

## Abstract

Many pathogenic microorganisms express fibronectin-binding molecules that facilitate their adherence to the extracellular matrix and/or entry into mammalian cells. We have previously described a *Borrelia recurrentis* gene, *cihC* that encodes a 40-kDa surface receptor for both, fibronectin and the complement inhibitors C4bp and C1-Inh. We now provide evidence for the expression of a group of highly homologues surface proteins, termed FbpA, in three *B. hermsii* isolates and two tick-borne relapsing fever spirochetes, *B. parkeri* and *B. turicatae*. When expressed in *Escherichia coli* or *B. burgdorferi*, four out of five proteins were shown to selectively bind fibronectin, whereas none of five proteins were able to bind the human complement regulators, C4bp and C1-Inh. By applying deletion mutants of the *B. hermsii* fibronectin-binding proteins a putative high-affinity binding site for fibronectin was mapped to its central region. In addition, the fibronectin-binding proteins of *B. hermsii* were found to share sequence homology with BBK32 of the Lyme disease spirochete *B. burgdorferi* with similar function suggesting its involvement in persistence and/or virulence of relapsing fever spirochetes.

## Introduction

*B. hermsii,* the causative agent of tick-borne relapsing fever is transmitted to humans through the bite of its infected argasid tick vector, *Ornithordoros hermsi* [1]. Rodents are the natural vertebrate hosts for *B. hermsii*, whereas *B. parkeri* may be pathogenic in horses and *B. turicatae* was shown to be pathogenic in dogs [2]–[4]. Patients suffering from relapsing fever were infected with those *Borrelia* species that occur in spirochete-infected ticks of the respective geographic areas [1], [2], [5]. Recently, two genomic groups of *B. hermsii,* termed I (GGI) and II (GGII) were identified. Isolates FRO and HS1 belonging to GGI and YOR to GGII were shown to be distinct from isolates of *B. turicatae* and *B. parkeri* spirochetes [6]–[8].

*B. hermsii* have evolved several strategies to persist in hosts and to evade innate and adaptive immunity, including antigenic variation, as seen for Vmp proteins [9]–[11] and specific binding, via cell surface receptors, of host derived complement regulators [12]–[15]. In fact, the surface bound complement inhibitor factor H (CFH) was shown to interfere with the alternative complement pathway by inhibiting complement activation via accelerating the decay of the C3 convertase and inactivating newly formed C3b [16]. Moreover, *B. hermsii* as well as other spirochetes, including *B. burgdorferi*, the causal agent of Lyme disease and the louse-borne relapsing fever spirochete *B. recurrentis*, were shown to specifically bind and exploit functional active host-derived proteases, e.g. human plasminogen, to facilitate immune evasion and/or persistence [12], [17]–[19].

We recently demonstrated that *B. recurrentis* express another outer membrane receptor for complement regulators of the classical and lectin pathway C4bp and C1-Inh, termed CihC, that also binds fibronectin, a human extracellular glycoprotein involved in microbial adhesion and colonization during the early phase of the infection process [20]–[22]. A related fibronectin and glycosaminoglycan binding protein of *B. burgdorferi,* BBK32, was shown to bind to endothelial cells *in vivo*, thereby initiating micro-vascular processes [23].

In this study we have cloned and characterized a group of highly homologous surface proteins of *B. hermsii*, *B. parkeri* and *B. turicatae,* which selectively bind fibronectin, but fail to interact with C4bp. The fibronectin-binding region was located to the central domain of the molecule. The outer surface location of FbpA and its putative role as an adhesion may enhance our understanding of the pathogenesis and persistence of relapsing fever spirochetes.

## Materials and Methods

### Bacterial strains and growth conditions

*B. parkeri* (isolate RML), *B. turicatae* (isolate 91E135), *B. hermsii* strains HS1, YOR and FRO (provided by Tom G. Schwan, Rocky Mountain Laboratories), *B. recurrentis* A1 and *B. duttonii* LA (provided by S. Cutler, University of East London) and Lyme disease spirochetes *B. burgdorferi* ZS7 and B313 as well as *B. garinii* ZQ1 and *B. afzelii* MMS were cultivated in BSK-H complete medium (Bio&SELL, Feucht, Germany) supplemented with 3% rabbit serum (PAN Biotech, Aidenbach, Germany) at 30°C [8], [24], [25]. B313 spirochetes harbor plasmids cp32-1, cp32-2, cp32-3, cp32-4, cp26 and lp7 exclusively and therefore lack expression of several major outer surface proteins, e.g. OspA, DbpA, BBK32, as well as several complement regulator-binding proteins BbCRASP-1 to -4 [8], [26]–[28]. Bacteria were harvested by centrifugation and washed with phosphate-buffered saline. The density of spirochetes was determined using dark-field microscopy and a Kova counting chamber (Hycor Biomedical, Garden Grove, CA). *Escherichia coli* JM109 were grown at 37°C in LB medium.

### Cloning of the *FbpA* genes, construction of expression plasmids and production of recombinant proteins

Whole-genome sequencing of *B. parkeri* DNA was performed in the Genome Sequencer FLX (454 Life Science/Roche) using FLX or FLX Titanium reagents according to the manufacturer's protocols and instructions [29]. A search for sequences homologues to *cihC* from *B. recurrentis* A1 produced a single match with an open reading frame of 1080 nucleotides, named *fbpA*. A 1.3-kb fragment containing the complete *fbpA* gene including approximately 100 bp upstream and downstream non-coding DNA was amplified by PCR from genomic DNA of *B. parkeri* strain RML by using primers BpF0 and BpR (Table 1). For cloning of *fbpA* from *B. turicatae*, primers BpF and BpR were used. A *B. hermsii* strain YOR genomic library was screened using a 500-bp fragment encompassing the 3′ end of the *fbpA* gene from *B. parkeri* strain RML as probe. A 1.5-kb clone containing *fbpA* and adjacent up- and down-stream sequences was identified, sequenced and selected for further analysis. The FbpA encoding genes from *B. hermsii* isolates were amplified by PCR using genomic DNA in combination with primers YORPromF and YORR (Table 1). Amplified DNA fragments were cloned into the vector pGEM-Teasy (Promega, Mannheim, Germany). The resulting plasmids were used as templates for construction of expression plasmids by PCR amplification. For recombinant full-length FbpA protein of *B. hermsii* isolates FRO, YOR and HS1, primers YORBam and YORR were used. For construction of C-terminal deletion mutants, these plasmids were digested with either restriction enzyme SpeI, EcoRV, and HincII in combination with HindIII. Blunt ends were created by treatment of the DNA with T4 DNA polymerase and the plasmids were ligated resulting in recombinant proteins FbpA(FRO)_20–279_, FbpA(FRO)_20–223_ and FbpA(FRO)_20–121,_ respectively. After digestion with restriction enzymes BamHI and PstI the amplified DNA fragments were ligated in frame into the His_6_-tag encoding sequence of vector pQE30Xa (Qiagen, Hilden, Germany). For expression of the His-tagged FbpA fusion proteins the plasmids were transformed in *E. coli* JM109 and recombinant proteins were purified as recommended by the manufacturer (Qiagen).

**Table 1:** Oligonucleotides used in this study.

Primer	Sequence (5′ to 3′)	purpose of use
YORR	GACTGTAAAATTGAGTCTATCTGTTG	amplification of *fbpA* genes of *B. hermsii* FRO, YOR, HS1
YORProm F	CTAATTCAAAATGCCCTATCTC	amplification of *fbpA* genes of *B. hermsii* FRO, YOR, HS1
YORBam	GTTTTATTGGATCCGACTTATTATTTG	generation of expression plasmids
YORPromF Bam	CTAATTCAAAATGCCGGATCCC	construction of pKFFS expression plasmids
BpF0	AGCTTTAGCCCTTCCCACATCAC	amplification of *fbpA* genes of *B. parkeri*
BpF	GATTTCTCTAAGGAGGTAACGATG	amplification of *fbpA* genes of *B. turicatae*
BpR	GACTATAAATGTAGCCTATCTTTGTTG	amplification of *fbpA* genes of *B. parkeri* and *B. turicatae*

### SDS-PAGE, ligand affinity blot and Western blot analysis

To prepare whole cell lysates *Borrelia* were centrifuged and washed three times with PBS. Cells were resuspended in BugBuster Master Mix (Merck) and lysed for 5–10 min at room temperature. Borrelial whole cell lysates (5 µg) or purified recombinant proteins (200 ng) were subjected to Tris/Glycine-SDS-PAGE under reducing conditions and transferred to nitrocellulose as previously described [30]. Briefly, the membrane was stained with 0.2% Ponceau S staining solution (Serva) for 5 min and rinsed with ultrapure water to verify that proteins were transferred. After transfer of proteins onto nitrocellulose, nonspecific binding sites were blocked using 5% (w/v) dried milk in TBS (50 mM Tris-HCl pH 7.5, 150 mM NaCl) for 2 h at room temperature (RT). Subsequently, membranes were rinsed twice in TBS and incubated for 1 h at RT with NHS (1:1 diluted in TBS) derived from blood donors that had never been exposed to relapsing fever spirochetes. Membranes were washed four times with 50 mM Tris-HCl pH 7.5, 150 mM NaCl, 0.2% Tween20 (TBST) and incubated for 1 h with peroxidase-conjugated anti-fibronectin (Southern Biotech) antibody followed by DAB treatment (Roche). For Western blot analysis, membranes were incubated for 1 h at RT with either anti-CihC mAb BR2 [20], anti-flagellin mAb LA21 or anti-C4bp antiserum (Quidel, San Diego). For detection of purified recombinant FbpA proteins, the anti-His_6_-tag (Calbiochem) antibody was employed. Following four washes with TBST, blot strips were incubated with a secondary peroxidase-conjugated anti-mouse IgG antibody (Dako, Glostrup, Denmark) for 1 h at RT. Detection of bound antibodies was performed using the enhanced chemiluminescence ECL Western blotting detection reagent and ECL Hyperfilms (GE Healthcare, Amersham).

### Construction of a shuttle vector for transformation of *B. burgdorferi* B313

The above-described *fbpA* genes including their native promoter regions of *B. hermsii* isolates YOR, FRO, and HS1 were subcloned by PCR using primers YORPromFBam and YORR into the pKFSS1 expression vector resulting in plasmids pKF-YOR, pKF-FRO and pKF-HS1, respectively. Transformation of *B. burgdorferi* B313 and characterization of transformants was previously described [19]. Expression of FbpA of transformed *B. burgdorferi* B313 was determined by Western blot and immunofluorescence analysis, using mAb BR2. High-passage, non-infectious *B. burgdorferi* strain B313 were grown in 100 ml BSK medium and harvested at mid exponential phase (10^8^ cells/ml). Electro-competent cells were prepared as described previously [26] with slight modifications. Briefly, 50 µl aliquots of competent *B. burgdorferi* strain B313 cells were electroporated at 12.5 kV/cm in 2-mm cuvettes with 10 µg of plasmid DNA. For control purpose *B. burgdorferi* strain B313 cells also were transformed with pKFSS1 vector alone. Cells were immediately diluted into 10 ml BSK medium and incubated without antibiotic selection at 30°C for 48 to 72 h. Bacteria were then diluted into 100 ml BSK medium containing streptomycin (75 µg/ml) and 200 µl aliquots were plated into 96-well cell culture plates [31] for selection of transformants. After three weeks, wells were evaluated for positive growth by color change of the medium, confirmed by dark-field microscopy for the presence of motile spirochetes. The *fbpA* gene of transformed *B. burgdorferi* B313 was detected by PCR using oligonucleotides YORPromFBam and YORR. Ectopic FbpA expression was analyzed using immunofluorescence microscopy and Western blot in combination with mAb BR2. In addition, ectopically expressed CihC was analyzed by ligand affinity blotting with regard to its capacity to acquire fibronectin.

### Immunofluorescence analysis

Spirochetes (1×10^7^) were washed with Tris buffer (30 mM Tris, 60 mM NaCl, pH 7.4) and incubated with mAb directed against CihC (mAb BR2) for 1 h at RT. Spirochetes were then washed with Tris buffer/0.1% BSA, spotted on coverslips and allowed to air-dry for 1 h. After methanol fixation, samples were dried for 15 min and incubated for 1 h in a humidified chamber with Cy3-labeled rabbit anti-mouse IgG (1/200, Dianova). Cells were visualized at a magnification of 1000x using a Nikon Eclipse 90i upright automated microscope and images were obtained using a Nikon DS-1 QM sensitive black and white CCD camera at a resolution of 0.133 µm/pixel.

### *In situ* protease treatment of spirochetes

Whole cells of non-transformed and transformed *B. burgdorferi* B313 were treated with proteases by modification of a method described previously [32]. Briefly, intact borrelial cells were incubated with either Proteinase K or trypsin and whole-cell protein preparations (10 µl) were separated using Tris/Tricine-SDS-PAGE via 4% stacking and 10% separating gels.

### Nucleotide sequence deposition

The *fbpA* gene sequences for *B. hermsii* strains YOR, FRO and HS-1, *B. parkeri* and *B. turicatae* have been deposited in the EMBL/GenBank databases under the following accession numbers HE983604, HE983605, HE983606, HE983607, and HE983608, respectively.

## Results

### Cloning and characterization of the receptor for fibronectin

**Figure 1:**
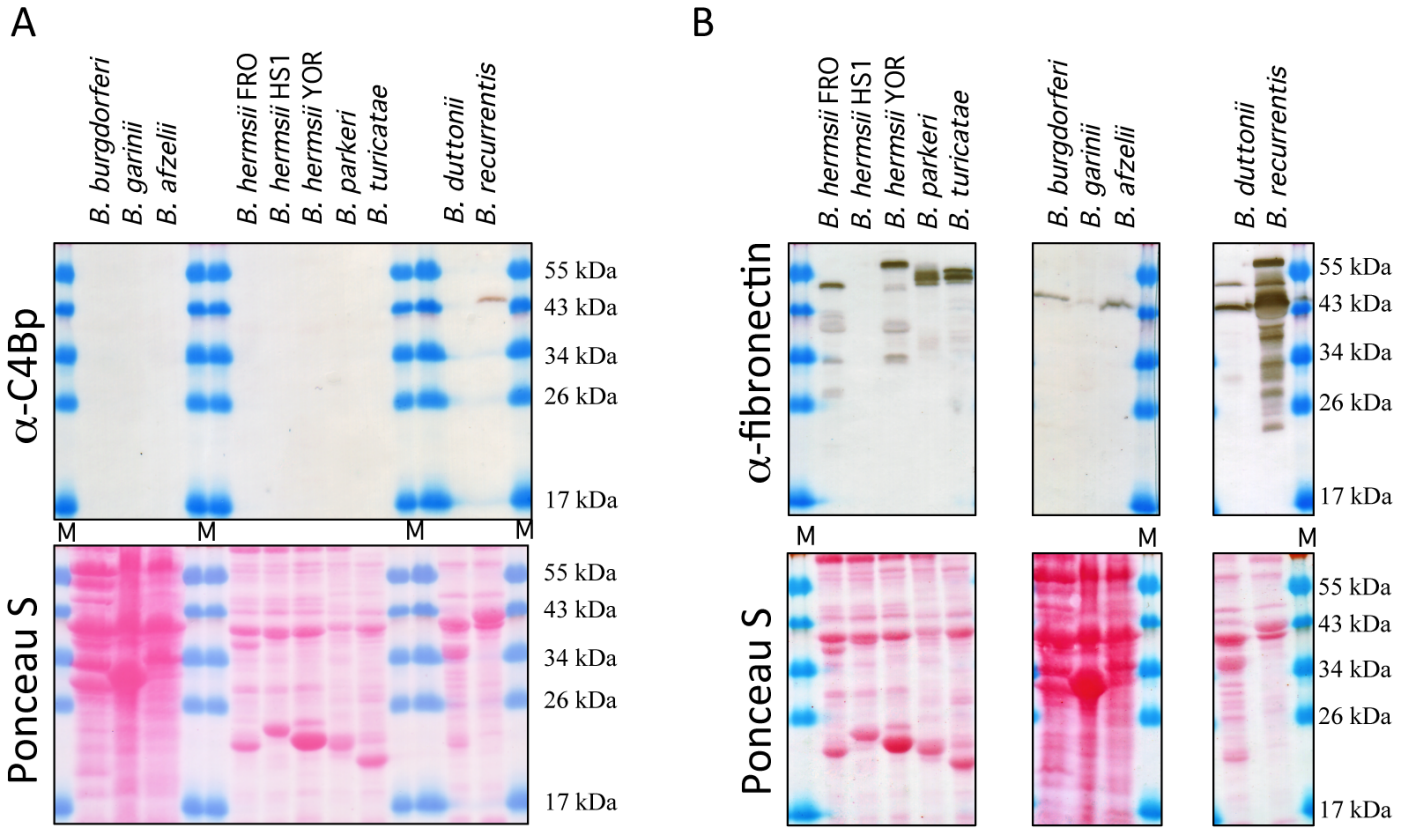
Binding of C4bp and fibronectin to spirochetal cell lysates. Whole cell lysates of tick-borne relapsing fever spirochetes *B. hermsii* YOR, FRO, and HS-1, *B. parkeri* RML, *B. turicatae* 91E135 and *B. duttonii* LA, the louse-borne spirochete *B. recurrentis* A1 and the Lyme disease organisms *B. burgdorferi* ZS7, *B. garinii* ZQ1 and *B. afzelii* MMS were separated by 13% Tris-Tricine SDS-PAGE, transferred to nitrocellulose membranes, and incubated with NHS. A) In the upper panel binding of C4bp was analyzed using mouse anti-C4bp IgG (Quidel, San Diego) and B) fibronectin binding was analyzed employing a specific goat immune serum (Southern Biotech). Lower panels indicate Ponceau S stain of bacterial cell lysates. Membranes were probed with anti-fibronectin peroxidase-conjugated IgG or with C4bp-specific antiserum followed by peroxidase-conjugated secondary antibody [20].

**Figure 2:**
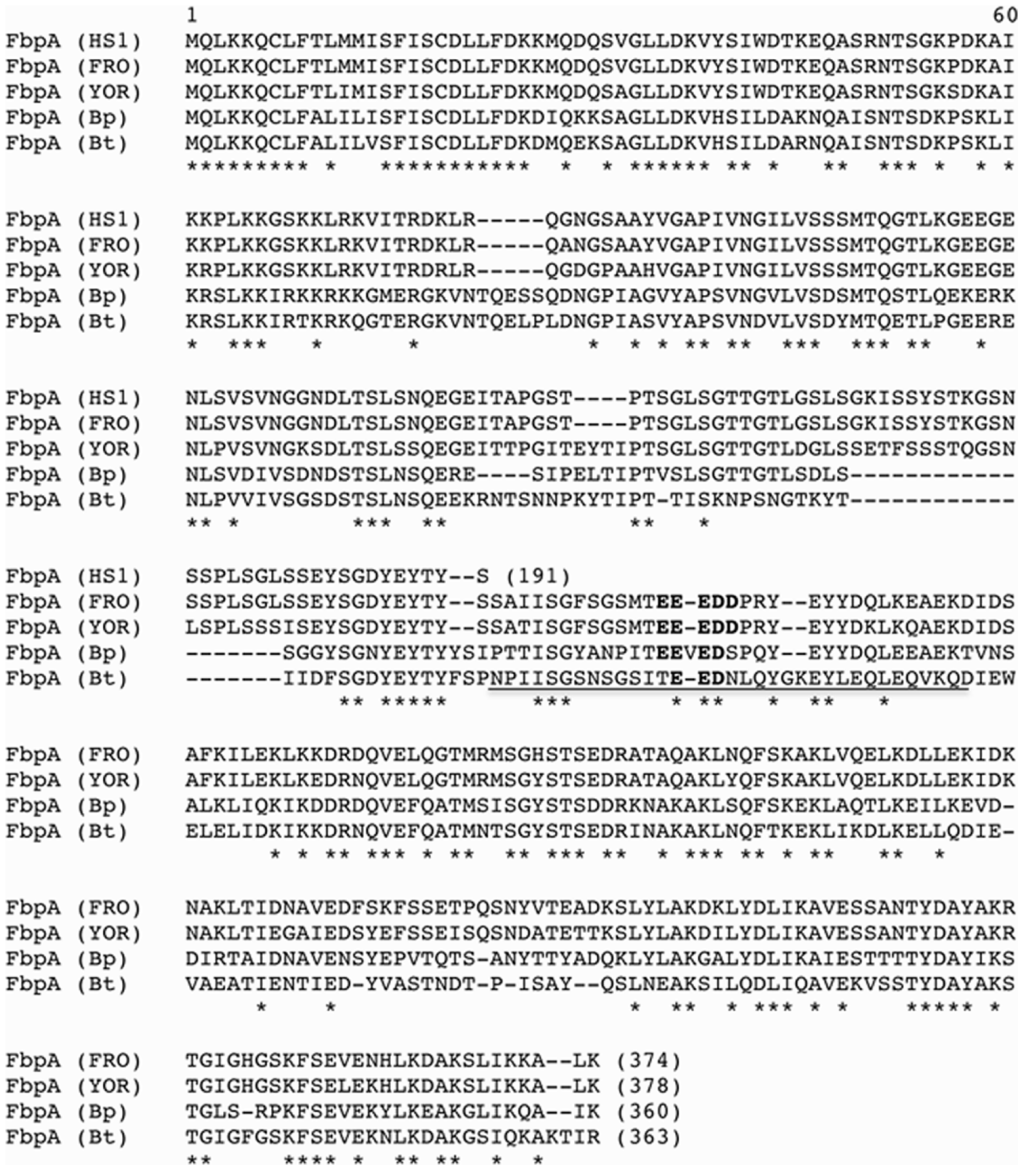
Alignment of FbpA sequences. Alignment of the deduced amino acid sequences of FbpA proteins from three isolates of *B. hermsii* (HS1, FRO, YOR) with the homologues FbpA proteins of *B. parkeri* and *B. turicatae*. Identical residues are marked with asterisks. A motif of acidic amino acid residues is indicated in bold. The putative fibronectin-binding region is underlined.

Previous experiments have shown that the louse-borne relapsing fever spirochete *B. recurrentis* and the African tick-borne spirochete *B. duttonii* specifically bind the human complement regulators C4bp and C1-Inh as well as fibronectin via their outer surface lipoprotein CihC. Screening for C4bp and fibronectin binding, employing cell lysates derived from North American tick-borne relapsing fever spirochete species *B. hermsii*, *B. parkeri* and *B. turicatae* either Ponceaus S stained (lower panels) or immunoblotted (upper panels) as depicted in Fig. 1 revealed that with the exception of *B. recurrentis* all other strains of tick-borne relapsing fever *Borrelia* as well as Lyme disease spirochetes (*B. burgdorferi* strain ZS7, *B. garinii* strain ZQ1 and *B. afzelii* strain MMS) failed to express C4bp-binding molecules (Fig. 1A). However, when spirochetal whole-cell lysates in combination with human serum and anti-fibronectin antibodies were employed, all but one (*B. hermsii* HS1) expressed dominant fibronectin-binding proteins in the range of 40–50 kDa (Fig. 1B). This finding is reminiscent of Lyme disease *B. burgdorferi* strains that express a fibronectin-binding protein, designated BBK32. Here we utilized a Roche/454-based whole genomic sequencing approach to identify the gene(s) encoding for complement regulator and/or fibronectin-binding protein(s) of a *B. parkeri* strain. To characterize the fibronectin-binding protein, named FbpA, of tick-borne relapsing fever spirochetes and to map the ligand-binding region, we PCR amplified homologues *fbpA* gene sequences encoding the FbpA of five different relapsing fever spirochetes. Most interestingly, the *fbpA* gene from *B. parkeri* is located 150 bp upstream of the *bpcA* gene (data not shown), which was recently shown to be located on a linear plasmid of approximately 200 kb [12], [13]. Each cognate *fbpA* gene and its appropriate flanking regions was PCR-amplified and inserted into pGEM-Teasy plasmid. N-terminal regions of the deduced proteins showed significant homologies to the signal peptides of other bacterial lipoproteins (Fig. 2). This motif includes two or three lysine residues near the N-terminus, a hydrophobic region, and a sequence with significant similarity to the consensus signal peptidase II cleavage sequence Leu(Ala, Ser)_−4_-Leu(Val, Phe, Ile)_−3_-Ile(Val, Gly)_−2_-Ala(Ser, Gly)_−1_-Cys_+1_ [33]. Using LipoP for prediction of lipoproteins of Gram negative bacteria a unique cleavage side for signal peptidase II was found between amino acid residue 18 and 19 suggesting lipidation at cysteine residue 19 of the fibronectin-binding proteins [34]. Amino acid alignment of the homologous proteins of all three *B. hermsii* strains detected a high degree of 87% sequence identity (Fig. 2). Moreover, the deduced FRO and HS1 amino acid sequences exhibited 99.5% identity at their N-terminal region encompassing amino acids 1 to 191 of FbpA. Most interestingly, *B. hermsii* strain HS1 showed a stop codon following amino acid residue 191 leading to a putative lipoprotein with a calculated molecular mass of only 20.2 kDa, whereas strain YOR and FRO encode full-length proteins of 41.5 kDa, which after processing are predicted to be 39.5 kDa in size (Fig. 2). Amino acid alignment of the fibronectin-binding proteins of *B. parkeri* and *B. turicatae* revealed about 64.2% sequence identity. Alignment of FbpA from these two isolates and the indicated *B. hermsii* strains revealed sequence identity ranging from 55–60%. As compared to the fibronectin-binding protein CihC of *B. recurrentis* and BBK32 of *B. burgdorferi*, FbpA molecules of *B. hermsii*, *B. parkeri* and *B. turicatae* revealed overall identity values of approximately 45% and 27%, respectively (data not shown).

**Figure 3:**
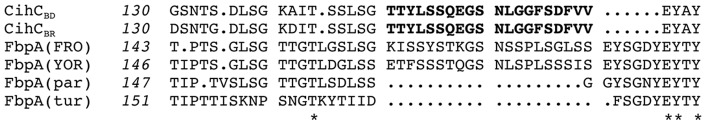
Alignment of partial sequences of FbpA and CihC. Alignment of FbpA amino acid sequences from two different *B. hermsii* (FRO and YOR), *B. turicatae* (tur) and *B. parkeri* (par) isolates with the putative central region of CihC_BR_ (*B. recurrentis*) and CihC_BD_ (*B. duttonii*) that bind human complement regulators, C4bp and C1-Inh (in bold). Identical residues are marked with asterisks.

We previously demonstrated that the putative C4bp-binding region of CihC of *B. recurrentis* (TTYLSSQEGSNLGGFSDFVV) is located to the central domain of the polypeptide [20]. This particular peptide region was missing from the FbpA sequence of *B. parkeri* and *B. turicatae.* In comparison to the FbpA molecules of *B. hermsii* FRO and YOR only 4 out of 20 amino acid residues of this region were conserved (Fig. 3). Thus it could be hypothesized that *B. parkeri*, *B. turicatae* and *B. hermsii* failed to bind C4bp due to significant differences in the homologues peptide segment of the respective FbpA molecules.

### Surface exposure and protease sensitivity of the receptor for fibronectin

**Figure 4:**
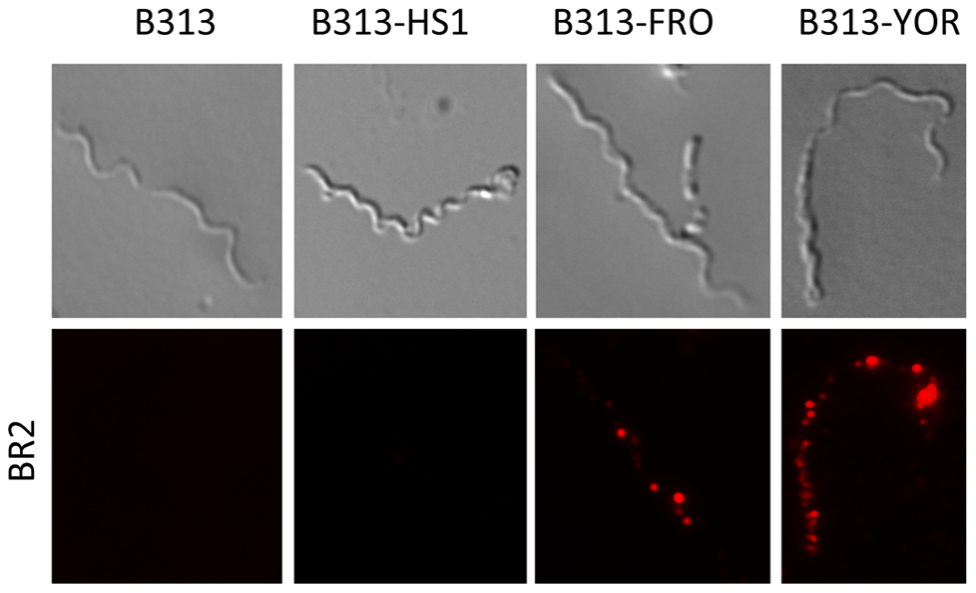
Surface localization of FbpA. Immunfluorescence analysis of *B. burgdorferi* B313 transformants harbouring the complete *fbpA* gene of *B. hermsii* strains HS1, FRO and YOR after incubation with a mAb specific for the C-terminal region of FbpA (BR2) followed by rabbit anti-mouse Cy3-conjugated IgG (lower panels). Corresponding differential interference contrast images are shown in the upper panels.

**Figure 5:**
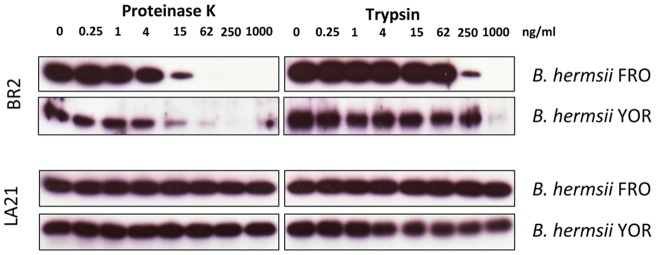
Surface localization of FbpA. Proteinase K and trypsin treatment affects surface expression of FbpA. *B. hermsii* FRO and YOR were incubated with the indicated concentrations of proteinase K and trypsin, lysed, immunoblotted, and probes with either anti-FbpA mAb BR2 (upper panel) or with anti-flagellin mAb LA21 (lower panel).

To determine whether FbpA molecules are exposed to the outer surface of *B. hermsii* immunofluorescence microscopy was performed. *B. burgdorferi* B313 strain, lacking fibronectin-binding protein BBK32 was transformed with the shuttle vector pKFSS1 containing the complete *fbpA* gene including its native promoter region. Several spirochetal clones expressing FbpA were selected for further analysis (data not shown). Transformed *B. burgdorferi* B313 spirochetes were incubated with the FbpA specific mAb BR2 followed by a Cy3-conjugated secondary antibody. Ectopic expression of *B. hermsii* YOR and FRO derived FbpA molecules on the outer surface of B313 showed a patchy distribution (Fig. 4). Due to the fact that the epitope recognized by mAb BR2 is located at the C-terminus of the full-length FbpA of *B. hermsii* (strains YOR and FRO) truncation of the C-terminal end from FbpA of strain HS1 completely abolished BR-2 binding activity. To further evaluate surface localization of FbpA we used Western blotting analysis. *B. hermsii* FRO and YOR spirochetes were pre-treated with either proteinase K or trypsin, lysed, separated by SDS-PAGE and assayed by Western blotting. As shown in Fig. 5 significant reduction of FbpA after 2 h of incubation with trypsin at concentrations ≥250 ng/ml, whereas treatment with proteinase K at low concentrations (≥62 ng/ml) resulted in complete degradation. Signal intensity observed for flagellin remained unchanged, indicating that periplasmic flagella were not affected by proteolytic digestion. These data strongly suggest that FbpA is exposed to the outer surface of *Borrelia*.

### Mapping the fibronectin-binding region of FbpA

**Figure 6:**
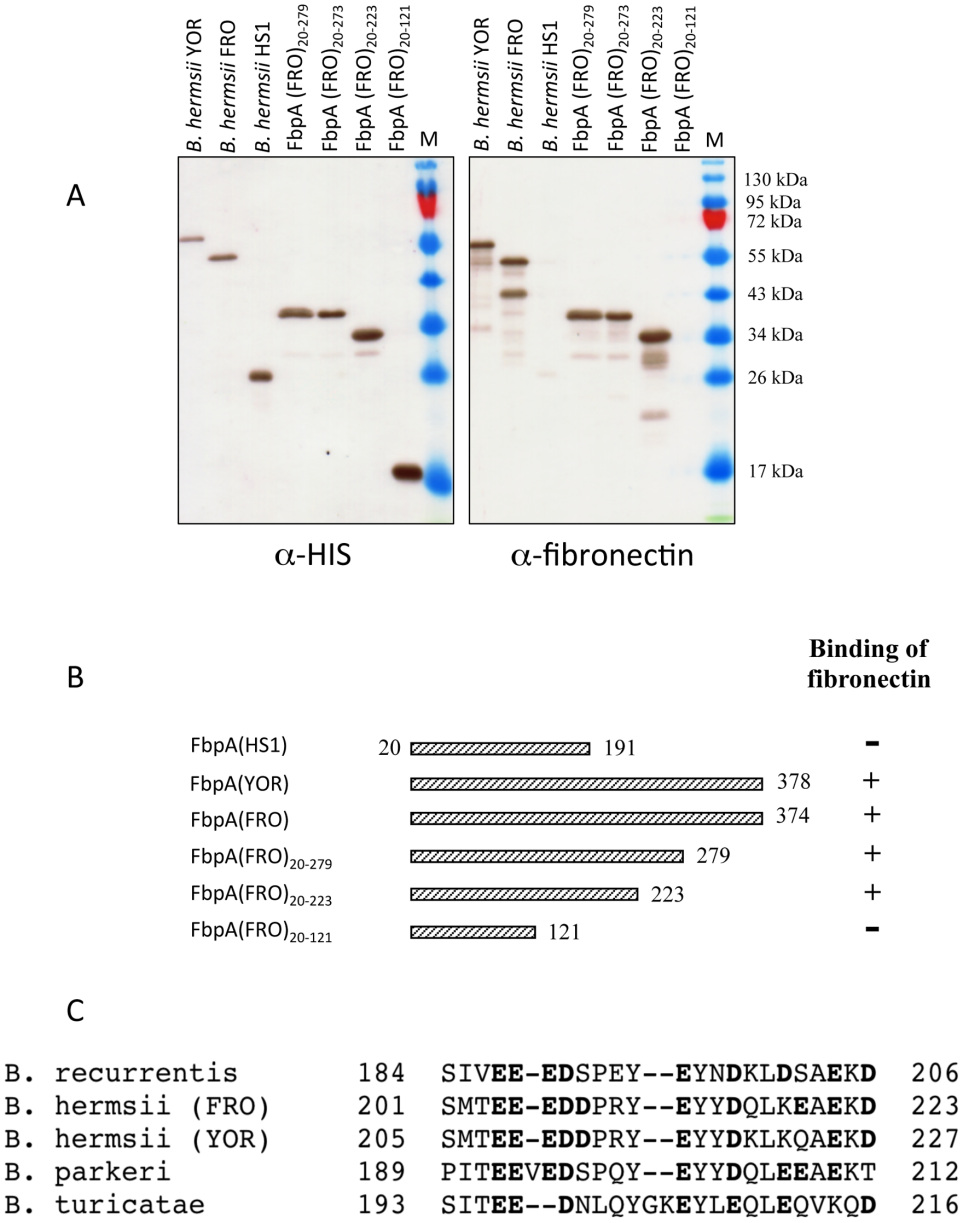
Mapping of the FbpA domain interacting with fibronectin. (A) *E.coli* lysates harboring recombinant FbpA of *B. hermsii* (strains HS-1, YOR and FRO) and the indicted deletion mutants of FbpA(FRO) were separated by SDS-PAGE and transferred to nitrocellulose. Membranes were incubated with anti-His-tag mAb (left panel). Fibronectin binding capability was analyzed by ligand affinity blotting utilizing normal human serum and with anti-fibronectin immune serum (right panel). (B) Diagrammatic representation of expressed recombinant FbpA and their binding characteristics for serum protein fibronectin as determined by ligand affinity blot analysis and ELISA. Numbers refer to amino acid residues. (C) Alignment of the predicted fibronectin-binding region (amino acid 184 to 200) of CihC of *B. recurrentis* and the respective FbpA domains of tick borne relapsing fever spirochetes. Acidic amino acid residues are indicated in bold.

To ascertain binding properties of the fibronectin-binding proteins we used C-terminal deletion mutants of FbpA. Variants of the encoding *fbpA* gene lacking the hydrophobic leader peptide and the indicated C-terminal regions were cloned and expressed as His-tagged fusion proteins in *E. coli*. Expression of each protein was confirmed by immunoblot analysis using His-tag specific antibody and all recombinant proteins exhibited the predicted size (Fig. 6A). Screening for fibronectin binding by ligand affinity blotting revealed that full-length FbpA of *B. hermsii* strains YOR and FRO as well as deletion mutants FbpA(FRO)_20–279_, and FbpA(FRO)_20–223_ bound fibronectin. No fibronectin-binding activity or immunoreactivity was observed with *E. coli* clone expressing the FbpA derived from strain HS1. Furthermore, deletion of 253 amino acids from the C-terminal end of FbpA(FRO) leading to mutant FbpA(FRO)_20–121_ completely abolished fibronectin-binding activity (Fig. 6A) suggesting that the fibronectin-binding region of FbpA is located to the central domain encompassing amino acids 191 to 223 (Fig. 6B). The alignment by Clustal analysis (HUSAR Bioinformatics Lab, genome.dkfz-heidelberg.de) of the predicted FhbA ligand binding domain from the indicated tick borne relapsing fever spirochetes and the putative fibronectin-binding region of *B. recurrentis* CihC revealed that the contiguous amino acid sequence motif EEED and relatively high number of acidic amino acid residues in the adjacent downstream region were highly conserved among all isolates tested in our study (Fig. 6C). This sequence characteristic has been noted for other bacterial fibronection-binding proteins [35], [36].

## Discussion

We have previously described a *B. recurrentis* cell surface lipoproteins, CihC, that binds both, host derived complement regulatory proteins C4bp/C1Inh and fibronectin [20]. Here we have identified and characterized a group of homologous proteins in the closely related American tick-borne relapsing fever spirochetes *B. hermsii*, *B. parkeri* and *B. turicatae*, that selectively bind human fibronectin but not C4bp. The putative high-affinity binding site for fibronectin was mapped to the central region of the receptor. The presence of adhesion molecules on the surface of relapsing fever spirochetes, capable to interact with components of the host's extracellular matrix suggests their involvement in processes of invasion, persistence and pathogenesis.

Spirochetes of the genus *Borrelia* causing Lyme borreliosis and relapsing fever express adhesins that bind fibronectin [37]–[39]. *B. burgdorferi* isolates express a 47-kDa fibronectin-binding adhesion, termed BBK32. The ligand-binding region of BBK32 from *B. burgdorferi* isolate B31 was associated with a 32-amino acids motif and solid phase binding experiments suggest that the unstructured N-terminal domain binds fibronectin [36], [38]. Further fibronectin-binding motifs were described for other bacterial proteins including fibronectin attachment protein and antigen 85b from mycobacteria, FnbpA from *Staphylococcus aureus*, and F1 protein from *Streptococcus pyogenes* [40]–[43]. Among these proteins, only the UR region of protein F1 from *S. pyogenes* shared significant sequence homology with the ligand-binding region of BBK32 with the amino acid sequence motif LSGESGEL representing the putative fibronectin binding domain [36].

We have previously reported on the identification of a singular outer membrane lipoprotein, CihC of *B. recurrentis,* with dual binding specificity for complement regulators and the extracellular matrix protein, fibronectin [20]. Although the biological relevance of simultaneous binding capacity of CihC for C4bp and fibronectin is unknown, one could speculate that it evolved due to the particularity of the transmission cycle of *B. recurrentis* between the body louse and human hosts [44].

Recent data provide evidence that fibronectin-binding proteins were found in *B. hermsii* and *B. turicatae* however, the respective genes encoding the putative surface proteins have not been identified yet [37]. We now cloned the *fbpA* genes of *B. hermsii* (YOR, FRO, and HS1), *B. parkeri* and *B. turicatae* strains. FbpA of *B. hermsii* HS1 shared 99% identity with the homologous protein of strain FRO. However, a stop codon was located at position 191 in the HS1 sequence resulting in a truncated FbpA molecule of 20-kDa. Thus, fibronectin-binding activity was undetectable in HS1 lysates suggesting that either FbpA was not expressed in *B. hermsii* HS1 or the fibronectin-binding site was localized to the missing C-terminal protein domain. However, a BLAST search of GenBank for sequences homologous to FbpA from isolate HS1 produced a match with the fibronectin-binding protein gene described for *B. hermsii* srain HS1 (accession number HQ698268). This HS1 gene was predicted to encode a 40-kDa protein. The discrepancy in the nucleotide sequence of both *B. hermsii* HS1 genes is unclear. Further studies using additional HS1 variants are urgently required to answer this question. In contrast, FbpA molecules of *B. hermsii* isolates FRO and YOR exhibit only 87% amino acid sequence identity supporting the notion that these two isolates belong to different genomic group, as has been demonstrated by Porcella et al. [7].

BLAST search of GenBank for sequences homologous to FbpA from *B. hermsii* isolates produces matches with the fibronectin-binding proteins BBK32 of *B. burgdorferi* (35% similarity) and CihC of *B. recurrentis* (54% similarity) [20], [38]. By aligning the 32-amino acid sequence of BBK32 from *B. burgdorferi* B31 with the FbpA sequences from three *B. hermsii* isolates overall sequence identities in the homologous ligand-binding motif only ranged from 22 to 25%. More importantly, the conserved amino acid sequence motif LSGESGEL in the ligand-binding region of BBK32 was not detectable in the FbpA amino acid sequences of all tested *B. hermsii* isolates, suggesting that in *B. hermsii* isolates different peptide motifs are involved in fibronectin-binding [36].

The putative peptide domain of FbpA relevant for fibronectin-binding was determined by using truncated C-terminal deletions mutants from strain FRO. Deletion mutants FbpA_20–279_ and FbpA_20–223_ fully retained fibronectin-binding activity. However, FbpA from HS1 and the C-terminal deletion mutant FbpA_20–121_ completely abolished fibronectin-binding activity, suggesting that amino acids 192 to 223 represent the fibronectin-binding region of FbpA. A striking feature of this sequence is a motif of acidic amino acid residues (EEED) within its central domain, which is also found in CihC. Moreover, deletion mutant analyses of CihC indicated that the fibronectin-binding region was located to this particular protein domain (unpublished data) suggesting that in accordance the respective EEEDD motifs of FbpA of *B. hermsii, B. parkeri* and *B. turicatae* represent the fibronectin-binding site [20], [35], [45]. However, we cannot exclude that additional fibronectin-binding sites, with properties similar to those of the indicated motif, are located on other spirochetal surface molecules. Thus, further studies are required to resolve this issue.

Adhesion to host tissues via fibronectin, heparin sulfate-glycosaminoglycan, collagen, integrins is a common feature in microbial invasion and pathogenesis [46], [47]. The expression profile and the fibronectin-binding activity of FbpA suggest that this surface molecule plays a pivotal role in the infection of relapsing fever spirochete, similar to that seen with BBK32 of the Lyme spirochete [36], [38], [48]-[51]. However, most of these studies have been performed in direct binding assays and tissue culture models *in vitro*. Therefore, the actual contribution of such interactions to dissemination and persistence in the host is still a matter of controversy. Most recently, the use of intravital microscopy in combination with fluorescent infectious stains of *B. burgdorferi* allowed to directly visualize interaction of spirochetes with endothelial cells of living murine hosts [23], [52], [53]. These investigations of *B. burgdorferi* dissemination *in vivo* have been performed in the presence of shear forces, microvascular endothelium and a functional immune system. The results obtained suggest that initiation of microvascular interactions required the *B. burgdorferi* protein BBK32, and host ligands fibronectin and glycosaminoglycans [23]. Thus, it could be speculated that fibronectin recruitment might be a feature of hematogenous dissemination of *Borrelia* species and may also have important implications for relapsing fever spirochetes. Further analysis of the precise mechanism underlying FbpA- and fibronectin-dependent dissemination under shear force condition will be required.

Taken together, our data shed some light on the molecular characterization of fibronectin binding molecules of the relapsing fever spirochetes *B. hermsii*, *B. parkeri* and *B. turicatae*. Elucidating the molecular and pathological processes underlying relapsing fever will be helpful to design novel strategies for therapeutic treatment and to develop potential vaccine candidates.
